# Public Health in an Era of Personal Health Records: Opportunities for Innovation and New Partnerships

**DOI:** 10.2196/jmir.1346

**Published:** 2010-08-10

**Authors:** Jason Bonander, Suzanne Gates

**Affiliations:** ^2^National Center for Public Health InformaticsCenters for Disease Control and PreventionAtlantaUSA; ^1^National Center for Chronic Disease Prevention and Health PromotionCenters for Disease Control and PreventionAtlantaUSA

**Keywords:** Personal health records, public health practice, informatics

## Abstract

In the near future, citizens will be able to control and manage their own health information through electronic personal health record systems and tools. The clinical benefits of this innovation, such as cost savings, error reduction, and improved communication, have been discussed in the literature and public forums, as have issues related to privacy and confidentiality. Receiving little attention are the benefits these will have for public health. The benefits and potential for innovation are broad and speak directly to core public health functions such as health monitoring, outbreak management, empowerment, linking to services, and research. Coupled with this is a new relationship with citizens as key partners in protecting and promoting the public’s health.

## Introduction

Personal health records (PHRs) have been a part of clinical care for decades. The most recognizable is a simple paper card that lists an individual’s immunization history. During the last 10 to 15 years, with the increasing use of technologies in clinical care and growth in electronic medical records (EMR), the PHR too has gone electronic. More recently, the electronic PHR has garnered increasing attention as a potential agent for citizen-centric health systems transformation [1,2], as a new personal tool for promoting health and health engagement [3-5], as a means for increasing efficiencies in appointment scheduling and medication refills, and as a means of improving the doctor-patient relationship [6,7]. At the time of this writing, a PHR can take multiple forms, ranging from stand-alone software for one’s computer to secure websites tethered to a specific practice or organization to general secure websites as platforms to integrate various kinds of health information. Each of these approaches supports different health needs such as tracking visits and costs, enabling secure email with physicians, scheduling appointments, refilling medications, and/or integrating clinical care. A subtle yet important difference in PHR offerings is the degree to which individuals have direct control over the data in their PHRs. Some PHRs offer “read only” access to one’s medical record, while others allow individuals to control who gets to view what within their record. There is considerable debate regarding the degree to which individuals should be able to control access to their health information and the forms that control may take [8,9].

Like EMRs, PHRs may also enhance the quality of care, improve doctor-patient communications, reduce the risk of medical errors, prevent unnecessary repetition of medical tests and procedures, and improve quality of life and health outcomes [2]. In addition, PHRs are beginning to allow individuals to add information such as weight or glucose readings, care plans, and customized applications with data accessible by mobile devices. Soon PHRs and personal health platforms will be able to seamlessly integrate with more robust mobile health devices, services, applications, and Web-based social networks. These capabilities can help individuals understand the information contained in their health records and relate it to daily activities and their environment, which could allow health and healthy living to become more integrated into daily living [5,10-11].

From the above description of PHR capabilities, both current and future, the public health opportunities with PHR systems start to gain clarity. However, public health has yet to significantly engage with the growing momentum despite significant opportunities. Potential benefits of PHRs to public health include providing information and resources, promoting healthier living, strengthening the continuum of care, and being a source of health monitoring data to supplement traditional public health activities such as surveillance and surveys. Research has shown that 75% of the US population would share personal health information with public officials to speed outbreak investigations and other public health activities given proper security and confidentiality measures [12]. Now is the time to think critically about public health’s role in the growing PHR movement: What potential do PHRs have in serving core public health functions?

## Public Health and Personal Health Records

A useful frame for public health activities is the set of ten essential public health services [13], which are:

- Monitoring health status to identify community health problems
- Diagnosing and investigating health problems and hazards in the community
- Informing, educating, and empowering people about health issues
- Mobilizing community partnerships to identify and solve health problems
- Developing policies that support individual and community health efforts
- Enforcing laws and regulations that protect health and ensure safety
- Linking people to needed personal health services
- Assuring a competent public health and personal health care workforce
- Evaluating effectiveness, accessibility, and quality of personal and population-based health services
- Conducting research to develop new insights and innovative solutions to health problems

The range of services performed by a given public health organization will depend upon whether it operates at the local, state, or federal level. Through these services, public health agencies seek to protect health, prevent disease and injury, promote healthy lifestyles and behaviors, respond to disasters, and assure accessible, high-quality health services. The innovation potential of PHRs will allow public health organizations to enhance each essential service, thereby building a much stronger and more effective public health system. Here, we discuss PHRs in the context of five of the essential services in the hopes of adding clarity to the issues and energy to the dialogue. This discussion also seeks to provide pointers to how we think some of these envisioned functions could become reality.

### Monitor Health

Monitoring population health status is a central public health activity. A variety of survey systems, such as the Center for Disease Control and Prevention’s National Health and Nutrition Examination Survey (NHANES) and National Health Interview Survey (NHIS), as well as state and national reporting systems, such as the Behavioral Risk Factor Surveillance System (BRFSS), Pregnancy Risk Assessment Monitoring System (PRAMS), and the National Electronic Disease Surveillance System (NEDSS), provide public health with the ability to assess health trends, identify and respond to emerging threats (eg, salmonella and E. coli) and guide development of interventions and policies that address serious health conditions (eg, obesity, smoking, and tuberculosis). These tools and reporting systems have been in operation for many years and remain essential to assessing health status, measuring disparities, and protecting the public’s health. However, PHRs hold the potential to improve health monitoring and may even foster entirely new approaches to this essential service. For example, through PHRs, individuals could easily, cheaply, and en masse share their health data anonymously with public health agencies, in essence creating “sentinel citizen” networks. Such networks could be both passive, where population data is anonymously analyzed in order to assess the prevalence of health issues, and active, where citizens elect to share targeted health information of interest to public health agencies. These approaches could enhance information gathering and reduce the resources necessary to support current health monitoring systems and further engage individuals as sentinels in protecting their own health and the health of their family, their community, and ultimately, the nation.

In a context where the public is actively and passively sharing health information with public health, this sharing also opens the door to a new kind of bidirectional relationship between individuals and public health, one not part of traditional public health monitoring activities. Currently, one of the few public health survey programs that has a bidirectional relationship with participants is NHANES. A routine part of the NHANES survey process is to synthesize survey findings and package them with a participant’s own health information as a “report of findings,” which is then sent through the mail to the NHANES participant. PHRs have the potential to streamline this process by offering to provide a participant’s health information in electronic form for integration with their other personal health data. By digitizing this process, the potential is created to establish a bidirectional communication channel for information sharing, future opt-in survey involvement, and so forth. While this may not be applicable to most of public health’s traditional survey activities, it does open a novel window on new ways to address public health’s mission.

Finally, a central issue with all health-monitoring activities is data quality. Even with current health monitoring activities, considerable time is spent managing data (ie, cleaning, integrating, and linking) to ensure it is of the highest quality. High quality data is the bedrock of evidence-based public health. PHRs have the ability to put some of these data management processes into the hands of individuals, allowing them to play a key role in maintaining the accuracy of their own personal health information. For example, individuals may identify and alert providers to gaps or inaccuracies in their personal health data ranging from personal characteristics, such as age, to past health events and current medications. Currently, health providers and administrators use such data primarily for administrative and reporting functions such as bill processing, service reimbursement, accreditation, and health monitoring. However, PHRs will allow individuals to monitor the quality of their health data/information and address data inaccuracies with their health providers, resulting in higher quality aggregated population health data reported at the local, state, and national levels. This example is not meant to address the validity or reliability of EMR or PHR data (an area in need of further research). Rather, we wish to highlight the effects that managing the quality of and controlling one’s personal health information could have on the quality of reported data (eg, vital statistics registries and immunization registries). Because clinical data are essential to understanding and addressing population health issues, improving the quality of these data could likely lead to development of more effective public health policies and interventions. A case in point would be birth registries and health information collected on the mother and baby around the birth event. The type and quality of data captured by current registries vary by state. What if a PHR could be created for an infant at birth by the mother through the birth registration process? What if a mother’s PHR could help provide information about her care and other data required at the birth registration (and if the mother did not have a PHR, offer her one through the auspices of the state health department)? These actions would not only strengthen birth registration content and information contained within PHRs, it would also impact the adoption of PHRs by tying them to birth registries. Further, mothers could provide data corrections back to the hospitals thereby compelling them to become more vigilant with regard to accuracy and the quality of health data in general as well as those data that are reported to public health.

### Investigate Health Issues

A key public health objective is to shorten the time it takes to identify and control potential health threats, and PHRs hold the potential to increase the speed and improve the efficiency and effectiveness of outbreak investigations. Combining PHRs with current and anticipated tools (eg, location-tagged mobile phone pictures linked to personal health information) could make it much easier for individuals to assist public health with real-time reporting during and after outbreaks and disasters. Individuals could voluntarily share personally identifiable or anonymous health data and information for a time-delimited period to rapidly aid public health officials in identifying and controlling an event. This would provide a convenient and critical feedback loop where individuals could get back information of value in return for sharing their information with public health and contributing to improved population health. Another expanded option would allow individuals to proactively combine their PHR data with regularly available health surveillance data in exchange for receiving timely, personalized, and localized public health alerts and notifications.

### Inform, Educate, and Empower

Driven by research and the changing incidence of health conditions, promotion of healthier behaviors is another of public health’s core functions. PHRs offer the potential for deeply tailored health promotion opportunities that could go far beyond current strategies to improve health behaviors. Strategies include, for example, Web-based programs to improve self-management of diabetes or support tobacco use cessation. While such strategies are still relevant, new tools could be designed to use existing PHR data to allow individuals to monitor their data and benchmark their health against those in their zip code, county, state, and nation. Putting nationally representative health data to work in this context could provide the impetus to create healthier communities and homes. Consider the potential of using PHRs to provide individuals with profiles of their neighborhood (eg, parks, bike trails, sidewalks, air quality, and grocery and retail stores within walking distance), along with recommendations tailored to an individual’s health characteristics. For example, could restaurant recommendations be made based on an individual’s BMI, weight maintenance goals, current dietary needs, the day’s level of physical activity, and geographical location?The potential benefits of converging personal health data with other information becomes a powerful tool for helping individuals incorporate healthy behavior into daily living.

### Link People to Services

Through PHRs, the process of connecting citizens with needed health services and resources could become more streamlined and tailored to local circumstances [14]. Using influenza vaccination as an example, the Advisory Committee on Immunization Practices (ACIP) has recommended that individuals at high risk for serious complications receive an annual vaccination (eg, children 6 months to age 19, individuals over 50, those with chronic conditions, and those caring for individuals at high risk for complications) [15]. With PHRs, alerts could be sent directly to individuals who should receive a vaccination, along with a localized map of nearby clinics offering this service with information on hours, languages spoken, and costs. By sending this information via a mobile device, public health could harness the power of immediacy. In this way, individuals could be reached not just where they live or work, but where they happen to be at any given time during the day. Taking this a step further, PHRs could enable individuals to immediately report receipt of a vaccination (with date, time, and geographic location) to public health monitoring systems, allowing for a more nuanced picture of vaccination behavior trends. Flu vaccination represents only an example of the multitude of service connections enabled by PHRs; other examples include cancer screening, other immunizations, mental health counseling, well-baby services, support groups, and so forth.

### Perform Research

Public health’s research agenda addresses key areas of disease and injury burden. Currently this research is carried through extramural partnerships with educational institutions, research institutes, and intramural projects. The emergence of PHRs creates new research opportunities and new ways to partner with the public in the research enterprise. For example, PHRs could support the ability to build longitudinal research panels that could support public health research on the myriad connections between population health and health care (eg, clinical preventive service use) and greater insight into disease prevention and control efforts and outcomes. Additionally, individual self-report data and information could be of great value in refining public health research. For example, research has already suggested that under the right circumstances, blood pressure monitoring could be more accurate when taken at home by educated patients using an approved calibrated device and sent in to the physician’s office as opposed to measurements taken in a clinical setting [16,17]. This example suggests an untapped continuum of qualitative to quantitative self-reported health information, which could be used to identify subtle aspects of contextual health behavior and develop more nuanced public health interventions, recommendations, and policies. Such innovation could result in a strengthened relationship with citizens as partners in the public health research process.

## More on Data Quality

There are important questions as to the quality and reliability of the data shared from PHRs. For example, how reliable is data that is entered by an individual into their PHR? How useful is this data for clinical or public health purposes? Might there be a continuum of data reliability and usefulness? For public health, this is not a trivial issue. Data validity and reliability are the bedrock of public health’s ability to create and recommend effective population-based policies and health interventions. As PHRs and other new health data sources become available, research will be needed to assess data quality, reliability, accuracy, and representativeness of each resource as it applies to the needs of public health [18].

## Public Health’s Role

There have been many cogent articulations and discussions of the significant challenges facing PHR adoption and use as well as policy levers to incent both individuals and organizations [2,19-23]. The challenges are not insignificant and at a minimum include being able to articulate the value of PHRs for individuals along the full wellness/sickness spectrum. The value and/or utility of PHRs outside of transaction-based functions (eg, appointment scheduling and medication refills), such as the potential benefits of PHRs for those managing chronic conditions [24], have not been sufficiently researched. However, as we work to increase our understanding of the uses and utility of PHRs, it would also serve the interests of public health to focus on issues related to data liquidity [25], personal health tools and platforms [5], general health literacy and education [26], and the ways in which these approaches and systems work to improve health outcomes and the quality of care as well as influence and streamline public health practice. Focusing energies and efforts in these areas should help prepare public health for what PHRs can become.

With these focal areas in mind, public health’s role vis-à-vis influencing policy gains some clarity. Data liquidity and secondary use of electronic health information are of great importance to public health. Supporting these interests, public health has been involved in the standards-setting processes for health information exchange, data standards that support enhanced health monitoring and electronic laboratory reporting. While indirect, these activities move the dial on getting electronic health information into the hands of health professionals, individuals, and families, enabling all aspects of health care systems to work smarter to protect health. Regarding personal health tools and health literacy, Healthy People has been an important national framework supporting these activities [27]. Born of the 1979 Surgeon General’s report, Healthy People [28] is now in its third decade of setting national health objectives that individuals, communities, organizations, and so on can use to develop programs to improve health. Components of *Healthy People 2010* included emphases on health literacy, health information quality, and improved general access to the Internet and Internet resources for all Americans. For *Healthy People 2020*, additional objectives have been proposed in the area of personalized health. Though still under development at the time of this writing, these include objectives such as increasing patient/provider interaction, increasing access to and use of personal health management tools, and increasing provider use of health information technology to improve population health. Each of these components plays a supporting role in the broader discussion of PHRs to build the systems and environmental variables tacked to improving personal health.

Lastly, there is significant room for public health’s contribution in the area of how PHRs work to improve health outcomes, improve the quality of care, and support public health practice. The majority of this paper has focused on how PHRs and related tools can be understood to fit within the context of public health services. However, as has been noted recently [29], though personal health tools appear to have some promise to improve health outcomes and the quality of care, the state of the research in this field is sorely wanting. Public health agencies could be contributing more to research at this intersection specifically focusing on how these tools improve care for at risk populations. In addition to research, there are questions of workforce readiness and capacity to take advantage of such tools. In order for the public health workforce to take advantage of PHRs and related tools, a number of issues need to be further explored and addressed. Both the Institutes of Medicine [30] and the Associated Schools of Public Health have been calling attention to a significant US public health workforce shortage by 2020. With an increasing prevalence of chronic conditions in the population, PHRs and personal health tools could be framed as an additional burden to an already burdened system or understood as an opportunity to streamline and/or change public health practices. Innovation is sometimes born of stressed systems. Irrespective of how these capabilities are understood, there is an underlying need for a larger public health workforce and one that is attuned to possibilities and capabilities of PHRs and personal health tools.

## Conclusion

We are witnessing the emergence and evolution of a health information economy the likes of which we have not seen before. Health information is increasingly updated and shared electronically, on a minute-by-minute basis. Investments made in the adoption of electronic medical records, most significantly supported through the American Recovery and Reinvestment Act, and increasing capabilities to securely exchange health information will only increase the speed at which this occurs. These trends and investments pave the way for new and innovative approaches and models for public health to extend its capabilities and achieve its mission. One of these will likely be PHRs. Here, we have highlighted the potential benefits of PHRs in terms of five essential public health services. Through PHRs and other sociotechnical innovations, public health has the opportunity to reimagine and reconsider the boundaries of traditional activities, such as health monitoring and threat investigations, and reconsider its traditional partnerships with individuals as public health information providers. PHRs also have the potential to provide tools to further strengthen other public health activities, such as educating and empowering people and linking them to needed health services. This is a fast-moving area, and policy considerations are currently being deliberated by national working groups such as the National Committee on Vital and Health Statistics [31]; data standards and models are being debated and proposed within national standards development organizations such as the Health Information Technology Standards Panel [32] and Health Level Seven; and applications are being rapidly developed to test these new waters—there are easily hundreds of health applications for the iPhone. For populations of particular concern for public health, PHR pilots are currently being conducted by the Centers for Medicare and Medicaid Services [33]. Data from these pilots will shed light on how these systems support the health needs of vulnerable and at risk populations. Further, with the potential increase in EMR adoption as stimulated through the American Recovery and Reinvestment Act of 2009, standardized health data will become readily available to populate PHRs [34]. There is much at stake in all of these activities and public health must have a direct and influential place at the table to ensure that these innovations will support core public health functions and benefit the protection and promotion of the population’s health.

## Abbreviations

BRFSS: Behavioral Risk Factor Surveillance System
EMR: electronic medical records
NEDSS: National Electronic Disease Surveillance System
NHANES: National Health and Nutrition Examination Survey
NHIS: National Health Interview Survey
PHR: personal health record
PRAMS: Pregnancy Risk Assessment Monitoring System
